# Efficacy and Hypoglycemia Profile of Once-weekly Insulin Icodec vs Once-daily Comparators Across Demographic Subgroups

**DOI:** 10.1210/clinem/dgaf168

**Published:** 2025-03-19

**Authors:** Ildiko Lingvay, Julie Krogsdahl Bache, Cyrus V Desouza, Mariana Fragão-Marques, Andrea Navarria, Shehla S Shaikh, André G D Vianna

**Affiliations:** Department of Internal Medicine/Endocrinology and Peter O’Donnell Jr. School of Public Health, University of Texas Southwestern Medical Center, Dallas, TX 75390, USA; Novo Nordisk A/S, 2860 Søborg, Denmark; Omaha VA Medical Center, University of Nebraska Medical Center, Omaha, NE 68105, USA; Novo Nordisk A/S, 2860 Søborg, Denmark; Faculty of Medicine of the University of Porto, 4200-450 Porto, Portugal; Novo Nordisk A/S, 2860 Søborg, Denmark; Saifee Hospital, Mumbai, Maharashtra 400004, India; Curitiba Diabetes Centre, Department of Endocrine Diseases, Hospital Nossa Senhora das Graças, Curitiba, Paraná 80810-040, Brazil

**Keywords:** ethnicity, race, demographic analyses, long-acting basal insulin, type 2 diabetes

## Abstract

**Objective:**

This post hoc analysis evaluated the impact of age, ethnicity, and race on efficacy and hypoglycemia outcomes with once-weekly insulin icodec (icodec) vs once-daily (OD) basal insulin comparators, leveraging data from the ONWARDS 1 to 5 phase 3a clinical trials.

**Methods:**

Efficacy and hypoglycemia outcomes were assessed within each trial in insulin-naive (ONWARDS 1, 3, and 5) and insulin-experienced (ONWARDS 2 and 4) adults (≥18 years) with type 2 diabetes across subgroups of age (<55, 55-64, and ≥65 years), ethnicity (Hispanic/Latino and non-Hispanic/Latino), and race (Asian, Black/African American, White, Other). The primary outcome was the change in glycated hemoglobin (HbA_1c_) from baseline to planned end of treatment. Other outcomes assessed included the achievement of HbA_1c_ <7% (<53 mmol/mol) without clinically significant or severe hypoglycemia and the number of clinically significant or severe hypoglycemic episodes.

**Results:**

Across all trials, the estimated treatment differences for change in HbA_1c_ and the odds ratios for achieving HbA_1c_ <7% (<53 mmol/mol) without clinically significant or severe hypoglycemia were similar across age, ethnicity, and race subgroups with icodec vs OD insulin (no statistically significant treatment by subgroup interactions were observed; *P* > .05 in all instances). Hypoglycemia rates were numerically low for both treatment groups and consistent across age, ethnicity, and race subgroups.

**Conclusion:**

The efficacy and hypoglycemia profile of icodec vs OD comparators was consistent across trial populations irrespective of age, ethnicity, or race.

Diabetes is a major global health challenge, affecting an estimated 536.6 million people, with approximately 90% of cases being type 2 diabetes (1, 2). A myriad of factors may affect or influence an individual's response to a given medication, including their age, ethnicity, and race, and the presence of comorbidities (3, 4). For example, populations of older individuals with type 2 diabetes are often highly heterogeneous, with widely varying durations of diabetes and time since diagnosis (5). Diabetes management in these individuals can be challenging, because older adults generally have a higher number of comorbidities than younger individuals, and advancing age is likely to be associated with declining cognitive function, which can increase the risk of severe hypoglycemia (3, 6). Consequently, there is a need for individualized treatment and diabetes management across different age groups (5).

Similarly, previous studies have shown increased type 2 diabetes prevalence, incidence, mortality, and diabetes complications in non-White populations compared with White populations (4, 7-10). Several differences across ethnic and racial populations have been described in relation to insulin sensitivity/resistance, insulin secretion and β-cell function, body composition, adiposity, body mass index (BMI), and environmental factors (eg, diet, physical activity, and access to healthcare) (4). Consequently, an individual's sensitivity and response to insulin therapy can vary depending on their ethnicity or race, which in turn will have implications for identifying the most appropriate insulin regimen and dosage (4, 11).

Given the differences in insulin response described here, it is important to be inclusive of different demographic subgroups when developing a novel insulin to understand how these variables may influence the response to treatment with regard to the efficacy and safety profile. Insulin icodec (icodec) is a basal insulin analog that is suitable for once-weekly administration (12). Icodec has been investigated, compared with once-daily (OD) comparators, in a global, phase 3a clinical development program comprising 6 trials (ONWARDS 1-6) (13-19). ONWARDS 1 to 5 evaluated the efficacy and safety of icodec across a range of clinical scenarios in individuals with type 2 diabetes (ClinicalTrials.gov: NCT04460885, NCT04770532, NCT04795531, NCT04880850, NCT04760626) (14-18). As the first once-weekly insulin, it is unclear what impact age, ethnicity, and race will have on the treatment response to icodec, and this therefore warrants evaluation.

Here, we describe a post hoc analysis of data from ONWARDS 1 to 5 that assessed, by trial, the efficacy and hypoglycemia profile of once-weekly icodec vs OD basal insulin comparators in adults with type 2 diabetes across different age, ethnicity, and race subgroups.

## Materials and Methods

### Trial Designs, Participants, and Treatment

Details of the ONWARDS 1-5 trial designs have been published; key points are summarized in this section (13-18). Briefly, ONWARDS 1 to 5 were randomized, controlled, multinational, multicenter, phase 3a trials that included insulin-naive (ONWARDS 1, 3, and 5) and insulin-experienced (ONWARDS 2 and 4) adults (≥18 years; no upper age limit) with type 2 diabetes. In total, 3765 participants were randomized across ONWARDS 1 to 5. All participants provided written informed consent before trial entry. A list of the relevant institutional ethics committees and institutional review boards for each trial is included in Supplementary Table 1 (20).

Participants were randomized (1:1) to either once-weekly icodec or OD comparator insulin (ONWARDS 1 and 4: insulin glargine U100; ONWARDS 2 and 3: insulin degludec; ONWARDS 5: insulin degludec, insulin glargine U100, or insulin glargine U300 chosen at the investigators’ discretion). In ONWARDS 4 (basal-bolus trial), participants in both treatment arms also received 2 to 4 daily injections of insulin aspart (17).

In insulin-naive participants (ONWARDS 1, 3, and 5), the starting dose of icodec was 70 U/week. For those previously treated with insulin (ONWARDS 2 and 4), the weekly icodec dose was the participant's pretrial insulin dose multiplied by 7; for the first injection only, a 50% 1-time additional dose was also administered. In ONWARDS 1 to 4, icodec and OD comparator insulin doses were titrated weekly based on target prebreakfast self-measured blood glucose values of 80 to 130 mg/dL (4.4-7.2 mmol/L). In ONWARDS 5, icodec titration was guided by a dosing guide app, whereas OD comparator insulin doses were titrated at the investigators’ discretion as per standard clinical practice (18).

In ONWARDS 1, 2, and 4, double-blinded continuous glucose monitoring (CGM) metrics were recorded using a CGM device at prespecified times throughout the treatment period (13-15, 17). CGM data were not collected in ONWARDS 3 or 5.

### Outcomes

In this post hoc analysis, key efficacy and hypoglycemia outcomes for icodec vs OD comparators within each trial were assessed across subgroups of age (<55, 55-64, and ≥65 years), ethnicity (Hispanic/Latino and non-Hispanic/Latino), and race [Asian, Black/African American, White, and Other (which included American Indian or Alaska native, and native Hawaiian or other Pacific Islander)]. Owing to only a few participants per trial being aged ≥75 years, participants aged ≥75 years were included in the ≥65 years subgroup. Consequently, a separate statistical analysis for participants aged ≥75 years was not conducted, but data relating to this population are included as a table in the Supplementary Materials (Supplementary Table 2) (20). Ethnicity and race were ascertained through participant self-identification during screening.

The primary outcome for all trials was the change in glycated hemoglobin (HbA_1c_) from baseline to planned end of treatment (EOT; ONWARDS 1: week 78; ONWARDS 2-4: week 26; ONWARDS 5: week 52).

CGM metrics, including the proportion of time in range [TIR; 70-180 mg/dL (3.9-10.0 mmol/L)], time above range [TAR; >180 mg/dL (>10.0 mmol/L)], and time below range [TBR; <54 mg/dL (<3.0 mmol/L)] were measured during the last 4 weeks of treatment in ONWARDS 1, 2, and 4 only.

For all trials, prespecified additional assessments included the achievement of HbA_1c_ <7% (<53 mmol/mol) at planned EOT without clinically significant (level 2) or severe (level 3) hypoglycemia in the previous 12 weeks. Clinically significant hypoglycemia was defined as a blood glucose level of <54 mg/dL (<3.0 mmol/L), confirmed by blood glucose meter; severe hypoglycemia was defined as hypoglycemia associated with severe cognitive impairment requiring external assistance for recovery.

For safety endpoints, the number of episodes of clinically significant hypoglycemia, severe hypoglycemia, and combined clinically significant or severe hypoglycemia from baseline to planned end of trial (ONWARDS 1: week 83; ONWARDS 2-4: week 31; ONWARDS 5: week 57) was reported.

Mean total weekly insulin dose during the last 2 weeks of treatment and change in body weight from baseline to planned EOT were also assessed.

### Statistical Analyses

Details of the statistical methods used to analyze data collected during ONWARDS 1 to 5 have been published; key details are summarized in this section (13-18).

Change in HbA_1c_, change in body weight from baseline to EOT, and weekly insulin dose during the last 2 weeks of treatment (log-transformed) were analyzed using an analysis of covariance model with treatment, region, subgroup, and treatment by subgroup interaction as fixed factors and baseline response as covariate (insulin dose as log-transformed baseline response was only used in ONWARDS 2 and 4).

The achievement of HbA_1c_ <7% (<53 mmol/mol) without clinically significant or severe hypoglycemia (composite assessment) was analyzed using a logistic regression model with logit link function with the same fixed factors as mentioned previously and baseline HbA_1c_ values as a covariate.

CGM endpoints (except TBR) were analyzed using an analysis of variance model with treatment, region, subgroup, and treatment by subgroup interaction as fixed factors.

TBR was analyzed using a negative binomial regression model (log link) on the number of recorded measurements below range. The model included treatment, region, subgroup, and treatment by subgroup interaction as fixed factors, with the logarithm of the total number of recorded measurements as an offset.

*P* values represented the test for no treatment interaction by age, ethnicity, or race subgroups, tested at a 5% 2-sided significance level. All statistical analyses were conducted using SAS software, version 9.4.

## Results

### Baseline Characteristics

Baseline characteristics by age, ethnicity, and race subgroups for the participants in ONWARDS 1 to 5 are summarized in Table 1. Although baseline characteristics were broadly balanced between treatment arms across the different subgroups, there were some notable observed trends. Compared with the other subgroups, individuals in the <55 years and Hispanic/Latino subgroups tended to have a higher baseline HbA_1c_ level, and individuals in the ≥65 years and Asian subgroups tended to have a lower baseline BMI. These differences in baseline characteristics occurred regardless of the treatment arm to which participants had been randomized. Overall participant baseline characteristics and participant disposition are shown in Supplementary Tables 3 and 4 (20).

**Table 1.** Baseline characteristics for the ONWARDS 1 to 5 participants by age, ethnicity, and race

	ONWARDS 1		ONWARDS 3		ONWARDS 5		ONWARDS 2		ONWARDS 4	
	Icodec	Glargine U100	Icodec	Degludec	Icodec + app	OD insulin analogs*^a^*	Icodec	Degludec	Icodec + aspart	Glargine U100 + aspart
Participants, n	492	492	294	294	542	543	263	263	291	291
Age subgroups, n (%)										
<55 years	154 (31.3)	154 (31.3)	113 (38.4)	96 (32.7)	169 (31.2)	165 (30.4)	51 (19.4)	41 (15.6)	80 (27.5)	81 (27.8)
55-64 years	179 (36.4)	178 (36.2)	97 (33.0)	105 (35.7)	190 (35.1)	198 (36.5)	94 (35.7)	108 (41.1)	109 (37.5)	103 (35.4)
≥65 years	159 (32.3)	160 (32.5)	84 (28.6)	93 (31.6)	183 (33.8)	180 (33.1)	118 (44.9)	114 (43.3)	102 (35.1)	107 (36.8)
Ethnicity subgroups, n (%)										
Hispanic/Latino	53 (10.8)	53 (10.8)	76 (25.9)	88 (29.9)	51 (9.4)	44 (8.1)	16 (6.1)	16 (6.1)	52 (17.9)	53 (18.2)
Non-Hispanic/Latino	439 (89.2)	439 (89.2)	203 (69.0)	190 (64.6)	490 (90.4)	499 (91.9)	247 (93.9)	247 (93.9)	239 (82.1)	237 (81.4)
Not reported	0 (0.0)	0 (0.0)	15 (5.1)	16 (5.4)	1 (0.2)	0 (0.0)	0 (0.0)	0 (0.0)	0 (0.0)	1 (0.3)
Race subgroups, n (%)										
Asian	129 (26.2)	145 (29.5)	80 (27.2)	85 (28.9)	28 (5.2)	19 (3.5)	86 (32.7)	110 (41.8)	95 (32.6)	93 (32.0)
Black/African American	10 (2.0)	17 (3.5)	9 (3.1)	6 (2.0)	24 (4.4)	28 (5.2)	11 (4.2)	12 (4.6)	13 (4.5)	8 (2.7)
Other	20 (4.1)	13 (2.6)	11 (3.7)	12 (4.1)	11 (2.0)	3 (0.6)	5 (1.9)	4 (1.5)	0 (0.0)	2 (0.7)
White	333 (67.7)	317 (64.4)	179 (60.9)	175 (59.5)	478 (88.2)	493 (90.8)	161 (61.2)	137 (52.1)	183 (62.9)	187 (64.3)
Not reported	NA	NA	15 (5.1)	16 (5.4)	1 (0.2)	0 (0.0)	NA	NA	0 (0.0)	1 (0.3)
Male, n (%)										
Age subgroups										
<55 years	97 (63.0)	81 (52.6)	66 (58.4)	65 (67.7)	104 (61.5)	88 (53.3)	32 (62.7)	24 (58.5)	39 (48.8)	47 (58.0)
55-64 years	107 (59.8)	99 (55.6)	63 (64.9)	63 (60.0)	103 (54.2)	121 (61.1)	58 (61.7)	56 (51.9)	60 (55.0)	50 (48.5)
≥65 years	91 (57.2)	83 (51.9)	56 (66.7)	56 (60.2)	102 (55.7)	104 (57.8)	72 (61.0)	60 (52.6)	55 (53.9)	53 (49.5)
Ethnicity subgroups										
Hispanic/Latino	31 (58.5)	24 (45.3)	37 (48.7)	50 (56.8)	21 (41.2)	22 (50.0)	7 (43.8)	7 (43.8)	23 (44.2)	26 (49.1)
Non-Hispanic/Latino	264 (60.1)	239 (54.4)	137 (67.5)	123 (64.7)	287 (58.6)	291 (58.3)	155 (62.8)	133 (53.8)	131 (54.8)	123 (51.9)
Race subgroups										
Asian	84 (65.1)	89 (61.4)	43 (53.8)	53 (62.4)	19 (67.9)	14 (73.7)	50 (58.1)	60 (54.5)	65 (68.4)	61 (65.6)
Black/African American	3 (30.0)	8 (47.1)	4 (44.4)	5 (83.3)	11 (45.8)	12 (42.9)	5 (45.5)	7 (58.3)	4 (30.8)	2 (25.0)
Other	13 (65.0)	5 (38.5)	2 (18.2)	6 (50.0)	7 (63.6)	2 (66.7)	4 (80.0)	2 (50.0)	0 (0.0)	2 (100.0)
White	195 (58.6)	161 (50.8)	125 (69.8)	109 (62.3)	272 (56.9)	285 (57.8)	103 (64.0)	71 (51.8)	85 (46.4)	84 (44.9)
Age, years, mean (SD)										
Age subgroups										
<55 years	47.18 (5.99)	46.95 (5.67)	47.19 (5.82)	47.26 (5.31)	46.51 (6.24)	47.32 (5.91)	47.71 (5.93)	48.73 (4.52)	46.27 (6.68)	47.40 (6.33)
55-64 years	59.79 (2.72)	59.74 (2.73)	59.51 (2.63)	59.32 (2.79)	59.30 (2.76)	59.54 (2.91)	59.66 (2.90)	59.83 (2.85)	60.40 (2.61)	59.63 (2.69)
≥65 years	69.75 (4.17)	69.33 (3.47)	69.74 (3.47)	69.37 (3.51)	70.66 (4.60)	70.31 (4.06)	70.81 (4.61)	70.21 (3.73)	69.39 (3.82)	69.65 (4.04)
Ethnicity subgroups										
Hispanic/Latino	56.00 (10.06)	57.32 (9.75)	54.22 (8.70)	56.08 (9.63)	56.31 (9.79)	56.02 (9.95)	60.25 (10.00)	57.50 (9.36)	55.94 (10.77)	56.77 (10.34)
Non-Hispanic/Latino	59.43 (9.99)	59.04 (9.86)	59.02 (10.46)	59.37 (9.49)	59.47 (10.85)	59.69 (10.13)	62.48 (9.78)	62.93 (8.27)	60.48 (9.83)	60.67 (9.68)
Race subgroups										
Asian	56.10 (11.26)	55.83 (10.84)	54.89 (10.64)	56.36 (9.69)	53.82 (9.51)	56.53 (11.33)	61.50 (9.90)	62.43 (8.57)	58.62 (10.96)	58.62 (11.10)
Black/African American	59.50 (8.00)	60.06 (6.72)	54.00 (8.94)	60.67 (8.14)	59.04 (11.66)	58.79 (10.74)	60.27 (8.38)	62.25 (8.23)	64.85 (7.44)	57.88 (10.91)
Other	55.50 (11.27)	56.77 (11.50)	50.91 (9.53)	58.42 (7.79)	58.45 (11.67)	44.67 (5.13)	58.80 (10.73)	61.50 (11.00)	NA	62.00 (5.66)
White	60.41 (9.24)	60.26 (9.14)	59.58 (9.68)	59.20 (9.70)	59.49 (10.76)	59.63 (10.03)	63.05 (9.80)	62.80 (8.34)	59.85 (9.77)	60.69 (9.23)
BMI, kg/m^2^, mean (SD)										
Age subgroups										
<55 years	31.01 (5.01)	31.02 (5.10)	30.03 (5.22)	29.50 (5.00)	34.11 (8.39)	35.06 (7.37)	30.75 (5.44)	30.19 (3.91)	31.20 (5.13)	29.33 (5.38)
55-64 years	29.68 (4.61)	29.81 (4.90)	29.64 (5.49)	29.55 (5.64)	32.21 (6.41)	32.87 (6.41)	30.63 (5.27)	29.38 (4.90)	31.26 (4.64)	30.69 (5.19)
≥65 years	29.32 (4.62)	29.64 (5.09)	29.92 (4.97)	28.64 (4.35)	31.49 (5.85)	31.11 (6.59)	28.11 (4.70)	28.61 (5.15)	29.28 (5.12)	29.79 (4.52)
Ethnicity subgroups										
Hispanic/Latino	30.72 (5.18)	29.85 (4.46)	30.55 (5.33)	30.27 (4.71)	34.21 (9.63)	31.32 (5.44)	32.09 (4.05)	31.43 (3.43)	32.22 (4.65)	31.61 (5.41)
Non-Hispanic/Latino	29.89 (4.73)	30.17 (5.12)	29.58 (5.23)	28.87 (5.14)	32.40 (6.65)	33.10 (7.05)	29.36 (5.22)	29.02 (4.94)	30.19 (5.03)	29.60 (4.87)
Race subgroups										
Asian	27.23 (4.53)	27.40 (4.53)	25.90 (3.71)	25.62 (3.23)	28.25 (5.63)	27.39 (4.49)	25.79 (4.29)	25.98 (3.65)	27.25 (4.23)	27.07 (4.12)
Black/African American	32.09 (2.74)	33.66 (4.99)	29.76 (5.14)	32.04 (4.22)	33.07 (9.60)	37.42 (9.66)	30.63 (5.62)	34.03 (3.44)	32.48 (3.92)	31.43 (3.53)
Other	30.44 (5.17)	30.64 (4.44)	31.59 (6.59)	30.78 (5.03)	31.49 (6.24)	33.93 (7.95)	33.13 (3.99)	31.66 (5.73)	NA	29.75 (1.01)
White	30.96 (4.49)	31.17 (4.80)	31.50 (4.86)	30.91 (4.87)	32.82 (6.87)	32.91 (6.68)	31.33 (4.56)	31.23 (4.34)	32.12 (4.63)	31.34 (4.92)
Diabetes duration, years, mean (SD)										
Age subgroups										
<55 years	8.13 (4.84)	8.90 (5.72)	8.73 (5.10)	8.74 (5.19)	8.60 (4.97)	9.05 (6.13)	11.09 (6.49)	11.29 (6.64)	12.87 (6.95)	12.13 (6.55)
55-64 years	12.38 (6.33)	11.76 (6.58)	11.75 (5.86)	11.17 (6.16)	11.61 (5.99)	11.29 (6.42)	15.33 (6.27)	16.39 (5.89)	17.29 (7.82)	15.76 (6.68)
≥65 years	14.15 (7.14)	13.58 (7.09)	13.71 (8.02)	14.67 (6.88)	15.14 (7.81)	15.42 (8.65)	19.86 (9.06)	19.47 (8.86)	22.69 (9.50)	20.04 (7.56)
Ethnicity subgroups										
Hispanic/Latino	11.50 (6.90)	13.77 (8.46)	9.93 (5.74)	10.50 (6.05)	10.45 (6.65)	10.65 (7.20)	16.60 (12.44)	18.83 (9.97)	16.84 (9.26)	14.25 (5.86)
Non-Hispanic/Latino	11.64 (6.64)	11.18 (6.47)	11.53 (6.88)	11.76 (6.78)	12.03 (6.92)	12.09 (7.63)	16.53 (8.06)	16.81 (7.77)	18.21 (9.05)	16.77 (7.94)
Race subgroups										
Asian	11.95 (6.95)	10.81 (6.64)	9.74 (6.08)	10.87 (6.44)	13.07 (7.15)	11.66 (7.95)	17.55 (8.75)	17.64 (8.01)	18.07 (8.92)	16.23 (8.28)
Black/African American	11.04 (6.20)	13.57 (7.75)	12.99 (5.78)	17.78 (5.10)	11.24 (6.23)	14.86 (10.01)	15.48 (6.45)	15.49 (12.60)	25.60 (10.44)	21.11 (9.28)
Other	13.06 (10.32)	14.27 (9.32)	9.01 (6.23)	12.95 (6.75)	13.71 (8.30)	3.32 (2.79)	17.33 (19.32)	17.00 (7.16)	NA	21.59 (3.03)
White	11.43 (6.29)	11.53 (6.61)	11.73 (6.83)	11.27 (6.59)	11.78 (6.91)	11.88 (7.41)	16.04 (7.81)	16.49 (7.38)	17.37 (8.87)	16.09 (7.26)
HbA_1c_, %, mean (SD)										
Age subgroups										
<55 years	8.64 (1.01)	8.62 (1.09)	8.75 (1.16)	8.59 (0.95)	9.26 (1.76)	9.14 (1.46)	8.36 (0.87)	8.32 (0.91)	8.45 (0.94)	8.52 (0.94)
55-64 years	8.47 (0.98)	8.38 (0.97)	8.49 (1.02)	8.65 (1.14)	8.90 (1.51)	8.92 (1.62)	8.19 (0.78)	8.08 (0.73)	8.29 (0.81)	8.46 (0.97)
≥65 years	8.42 (0.98)	8.34 (0.99)	8.35 (1.13)	8.19 (0.86)	8.74 (1.57)	8.61 (1.34)	8.07 (0.72)	8.04 (0.75)	8.16 (0.83)	8.01 (0.68)
Ethnicity subgroups										
Hispanic/Latino	8.72 (1.09)	8.57 (1.16)	9.09 (1.32)	8.61 (1.04)	10.18 (1.70)	9.96 (1.91)	8.38 (0.87)	8.44 (1.04)	8.39 (0.84)	8.27 (0.85)
Non-Hispanic/Latino	8.48 (0.98)	8.43 (1.00)	8.36 (0.98)	8.40 (0.98)	8.83 (1.56)	8.79 (1.42)	8.15 (0.77)	8.08 (0.75)	8.27 (0.86)	8.32 (0.91)
Race subgroups										
Asian	8.35 (1.00)	8.36 (1.08)	8.20 (0.91)	8.41 (0.95)	8.82 (1.30)	9.39 (1.40)	8.04 (0.68)	7.99 (0.72)	8.32 (0.94)	8.43 (0.95)
Black/African American	8.93 (0.80)	8.62 (0.93)	9.21 (1.12)	8.55 (1.91)	9.86 (2.22)	9.02 (1.62)	8.15 (0.91)	8.04 (0.82)	8.35 (0.59)	8.56 (0.98)
Other	8.43 (0.70)	8.55 (1.26)	9.19 (1.35)	8.63 (1.50)	8.58 (1.27)	8.07 (0.23)	7.96 (0.61)	8.43 (1.44)	NA	7.80 (1.13)
White	8.55 (1.01)	8.47 (0.99)	8.64 (1.16)	8.48 (0.95)	8.93 (1.60)	8.86 (1.49)	8.24 (0.81)	8.18 (0.79)	8.27 (0.84)	8.25 (0.86)

The “Other” race subgroup included American Indian or Alaska native, and native Hawaiian or other Pacific Islander.

Abbreviations: aspart, insulin aspart; BMI, body mass index; degludec, insulin degludec; glargine U100, insulin glargine U100; glargine U300, insulin glargine U300; HbA_1c_, glycated hemoglobin; icodec, insulin icodec; NA, not applicable; OD, once daily.

^a^In ONWARDS 5, participants in the OD comparator arm received OD degludec, glargine U100, or glargine U300 at the investigators’ discretion. Across trials, 174 participants were aged ≥75 years at baseline. Baseline characteristics for the 97 participants aged ≥75 years who received icodec (30-90% of whom were male) are as follows: mean baseline BMI range of 27.97 to 29.42 kg/m^2^, a mean diabetes duration range of 15.7 to 27.9 years, and mean baseline HbA_1c_ range of 7.95% to 8.88% (63.4-73.6 mmol/mol). Baseline characteristics for the 77 participants aged ≥75 years who received OD comparators (46-78% of whom were male) are as follows: mean baseline BMI range of 27.42 to 31.71 kg/m^2^, a mean diabetes duration range of 15.8 to 22.9 years, and mean baseline HbA_1c_ range of 7.99 to 8.53% (63.9-69.7 mmol/mol).

### Efficacy Endpoints

#### Change in HbA_1c_

**Figure 1.**
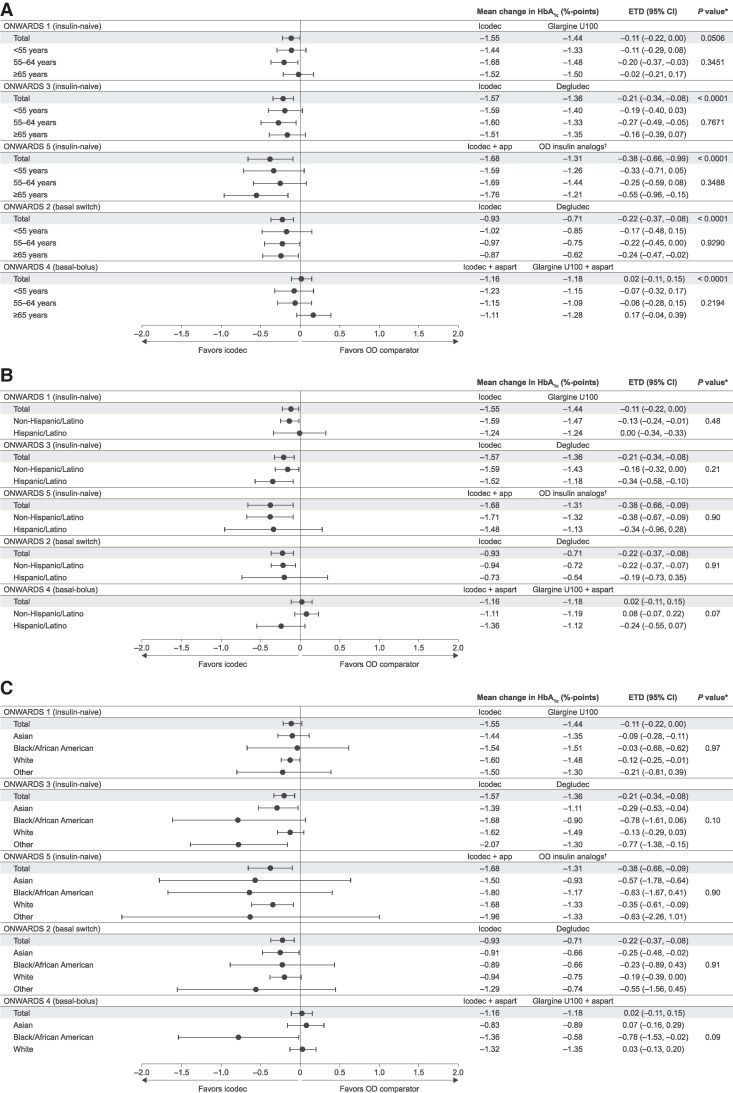
Estimated treatment differences in change in HbA_1c_ (percentage points) from baseline to planned EOT by (A) age, (B) ethnicity, and (C) race. Planned EOT: ONWARDS 1, week 78; ONWARDS 2-4, week 26; ONWARDS 5, week 52. Mean change in HbA_1c_ is the estimated change. The “Other” race subgroup included American Indian or Alaska native, and native Hawaiian or other Pacific Islander. *The *P* value is for test of no treatment interaction by subgroup. Modeled using an analysis of covariance model with treatment, region, subgroup, treatment by subgroup interaction, and, if applicable, additional relevant factors as fixed factors and baseline response as covariate. ^†^In ONWARDS 5, participants in the OD comparator arm received OD degludec, glargine U100, or glargine U300 at the investigators’ discretion. Abbreviations: Aspart, insulin aspart; degludec, insulin degludec; EOT, end of treatment; ETD, estimated treatment difference; glargine U100, insulin glargine U100; glargine U300, insulin glargine U300; HbA_1c_, glycated hemoglobin; icodec, insulin icodec; OD, once daily.

In ONWARDS 1 to 5, there were no statistically significant treatment by subgroup interactions for change in HbA_1c_ from baseline until planned EOT across age, ethnicity, or race subgroups. A numerically higher (ie, nonstatistically significant) reduction in HbA_1c_ was generally seen with icodec vs OD comparators across age, ethnicity, and race subgroups in ONWARDS 1 to 3 and 5; in ONWARDS 4, a similar reduction in HbA_1c_ was generally observed with icodec vs the OD comparator across subgroups, except for in the ≥65 years subgroup, in which a numerically higher reduction was seen with OD comparator (Fig. 1).

#### TIR, TAR, and TBR

Based on CGM data from ONWARDS 1, 2, and 4 (icodec, n = 1046; OD comparator, n = 1046), the estimated treatment differences (icodec vs OD comparator) for TIR and TAR were consistent across age, ethnicity, and race subgroups in ONWARDS 1 from week 74 to week 78, and from week 22 to week 26 in ONWARDS 2 and 4 [Supplementary Fig. 1, Supplementary Table 5 (20)]. There were no statistically significant treatment by subgroup interactions for percentage of TIR or TAR by age, ethnicity, or race.

Regarding the estimated treatment ratios for TBR from week 74 to week 78 (ONWARDS 1) and week 22 to week 26 (ONWARDS 2 and 4), there were also no consistent trends across age, ethnicity, and race subgroups in any of the trials [Supplementary Fig. 1, Supplementary Table 5 (20)], with no statistically significant treatment by subgroup interactions for ratio of TBR by age, ethnicity, or race subgroup.

#### Achievement of HbA_1c_ <7% (<53 mmol/mol) without clinically significant or severe hypoglycemia

**Figure 2.**
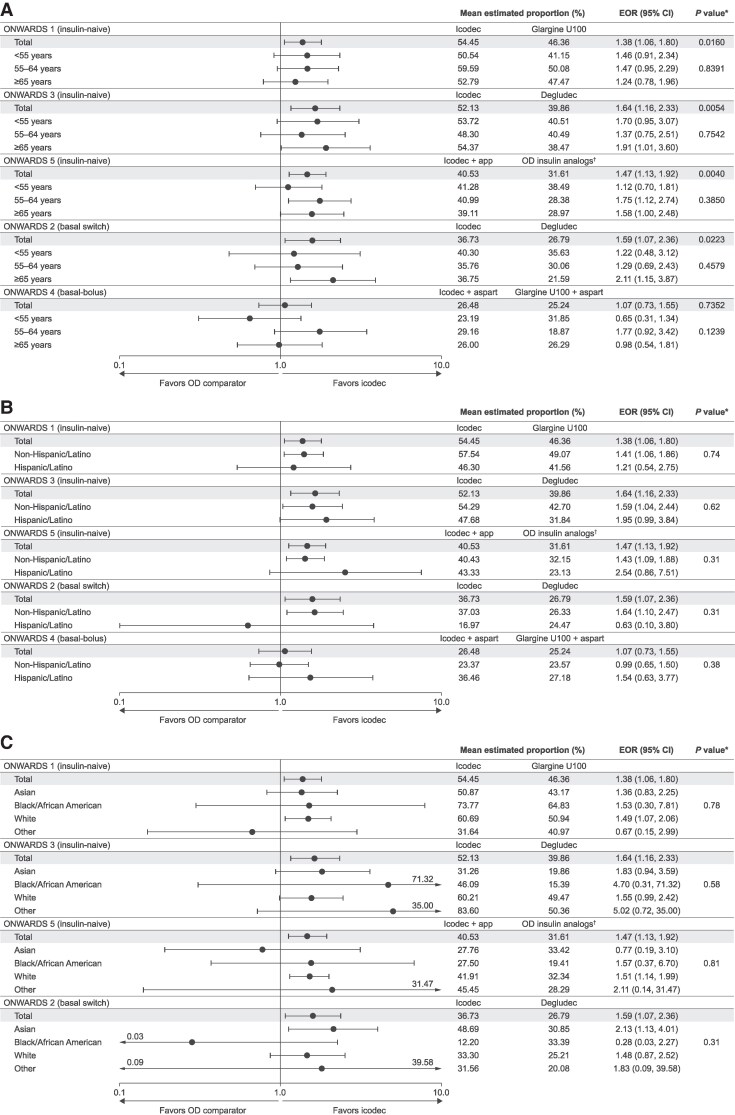
Estimated odds ratios for the achievement of HbA_1c_ <7% at planned EOT without clinically significant or severe hypoglycemia in the previous 12 weeks by (A) age, (B) ethnicity, and (C) race. Planned EOT: ONWARDS 1, week 78; ONWARDS 2-4, week 26; ONWARDS 5, week 52. Clinically significant hypoglycemia: blood glucose of <54 mg/dL (<3.0 mmol/L), confirmed by blood glucose meter. Severe hypoglycemia: hypoglycemia associated with severe cognitive impairment requiring external assistance for recovery. The “Other” race subgroup included American Indian or Alaska native, and native Hawaiian or other Pacific Islander. *The *P* value for treatment interaction by subgroup was calculated using a logistic regression model (logit link) with treatment, region, subgroup, treatment by subgroup interaction, and, if applicable, additional relevant factors as fixed factors and the baseline HbA_1c_ value as covariate. ^†^In ONWARDS 5, participants in the OD comparator arm received OD degludec, glargine U100, or glargine U300 at the investigators’ discretion. Abbreviations: Aspart, insulin aspart; degludec, insulin degludec; EOR, estimated odds ratio; EOT, end of treatment; glargine U100, insulin glargine U100; glargine U300, insulin glargine U300; HbA_1c_, glycated hemoglobin; icodec, insulin icodec; OD, once daily.

In ONWARDS 1 to 5, no statistically significant differences were seen in the treatment by subgroup interactions across the different age, ethnicity, and race subgroups concerning this composite assessment (Fig. 2).

Across all trials, the odds of achieving HbA_1c_ <7% (<53 mmol/mol) without clinically significant or severe hypoglycemic episodes in the previous 12 weeks at planned EOT were numerically higher for icodec vs OD comparators irrespective of age, ethnicity, or race subgroup (Fig. 2), although different trends in the odds of achieving this target were seen in the “Other” race subgroup (ONWARDS 1), Asian subgroup (ONWARDS 5), Hispanic/Latino subgroup (ONWARDS 2), and Black/African American subgroup (ONWARDS 2). In ONWARDS 4, the trends either favored icodec or presented similar odds of achieving the target between treatments, except for in the <55 years subgroup. Statistical analysis was not performed for race subgroups in ONWARDS 4 owing to the Black/African American (n = 10) and “Other” (n = 20) subgroups comprising too few participants.

### Safety Endpoints

#### Hypoglycemia rates

In ONWARDS 1 to 3 and 5, rates of combined clinically significant or severe hypoglycemic episodes were low in both treatment arms and generally consistent across all age, ethnicity, and race subgroups [Supplementary Tables 6 and 7 (20)], except for the Black/African American subgroup in ONWARDS 2 and 3, in which the higher number of events observed in the icodec arm were reported in 2 participants and 1 participant, respectively. In ONWARDS 4 (basal-bolus), rates of combined clinically significant or severe hypoglycemia were numerically higher than those in ONWARDS 1 to 3 and 5 but were consistent across subgroups, and there were no clinically relevant differences between treatment arms.

Rates of severe hypoglycemic episodes were very low across all trials. Across <55, 55 to 64, and ≥65 years age subgroups, 4, 3, and 1 severe hypoglycemic episodes were reported, respectively, for icodec, and 7, 5, and 6, respectively, for OD comparators (ONWARDS 1-5). For the Hispanic/Latino and non-Hispanic/Latino ethnicity subgroups, 0 and 8 severe hypoglycemic episodes, respectively, were reported for icodec, and 0 and 17, respectively, for OD comparators (ONWARDS 1-5). Finally, across race subgroups, 2 and 6 severe hypoglycemic episodes were reported in the Asian and White subgroups, respectively, for icodec, whereas 2, 14, and 1 were reported in Asian, White, and Black/African American subgroups, respectively, for OD comparators (ONWARDS 1-5). In participants receiving icodec, no severe hypoglycemic episodes were reported in ONWARDS 2, 3, and 5 across all age, ethnicity, and race subgroups.

#### Insulin dose during the last 2 weeks of treatment

No statistically significant treatment by subgroup interactions were observed in the mean total weekly insulin dose for icodec vs OD comparators by age, ethnicity, or race subgroup during the last 2 weeks of treatment in ONWARDS 1 to 5 [Supplementary Table 8 (20)], except for the age subgroup analysis in ONWARDS 2, in which a statistically significant treatment by subgroup interaction was observed [icodec/OD comparator estimated treatment ratio (95% confidence interval): <55 years, 0.86 (0.70, 1.04); 55-64 years, 1.23 (1.07, 1.40); ≥ 65 years, 1.08 (0.95, 1.22); *P* = .01].

#### Change in body weight

There were no statistically significant treatment by subgroup interactions in change in body weight observed from baseline to EOT by age, ethnicity, or race subgroup in any of the trials except for ONWARDS 2, in which a statistically significant treatment by race subgroup interaction was observed [icodec/OD comparator treatment ratio (95% confidence interval) for change in body weight: Asian, 1.21 (−0.22, 2.64); Black/African American, −0.70 (−4.61, 3.21); White, 2.40 (1.18, 3.62); Other, −5.91 (−12.17, 0.35); *P* = .03], with White participants experiencing a greater weight gain with icodec than with OD comparator [Supplementary Table 9 (20)].

## Discussion

This post hoc subgroup analysis of data from the ONWARDS 1 to 5 clinical trials suggests that the efficacy and hypoglycemia profile of icodec vs OD comparators remained generally consistent in insulin-naive and insulin-experienced adults with type 2 diabetes irrespective of age, ethnicity, or race subgroup. Overall, efficacy and hypoglycemia outcomes by age, ethnicity, and race were consistent with the results observed in the total ONWARDS 1 to 5 trial populations, with some exceptions discussed later.

Treatment differences for change in HbA_1c_ broadly favored icodec over OD comparators numerically across age, ethnicity, and race subgroups in insulin-naive and basal-only therapy individuals. In ONWARDS 4, the trend in change in HbA_1c_ observed in the ≥65 years subgroup was different from that seen in the overall trial population, numerically favoring the OD comparator. In the remaining ONWARDS 4 subgroups, estimates were near the reference line, which is in accordance with the primary analysis. Notably, there were no statistically significant interactions observed between treatment and age, ethnicity, or race subgroups for change in HbA_1c_ from baseline, suggesting that the efficacy of icodec observed in the total trial populations applies to each of the analyzed subgroups.

In ONWARDS 1 to 3 and 5, the odds of achieving an HbA_1c_ <7% (<53 mmol/mol) at planned EOT without clinically significant or severe hypoglycemia during the previous 12 weeks were numerically higher for the icodec arm than for the OD comparator arm, and the respective odds ratios were similar across age, race, and ethnicity subgroups. These findings were consistent with the results observed in the overall trial populations. Similarly, in ONWARDS 4, the odds of achieving the composite assessment were consistent with those from the overall trial population (no difference observed between treatment arms), with the exception of the <55 years subgroup, which presented a trend favoring the OD comparator, and the 55 to 64 years and Hispanic/Latino subgroups, both of which showed a trend favoring icodec. These results might be due to a real age/ethnic difference in response to icodec treatment or, more likely, an artifact due to a smaller number of participants in these subgroups than in other subgroups.

The rates of clinically significant or severe hypoglycemia were low (less than 1 event per patient-year of exposure) in both the icodec and OD comparator treatment arms in the overall trial population and generally consistent across subgroups in both treatment arms for ONWARDS 1 to 3 and 5. Rates of combined clinically significant or severe hypoglycemic episodes in ONWARDS 4, although consistent across demographic subgroups, were numerically higher than in the other ONWARDS trials analyzed, which may be attributable to participants in ONWARDS 4 receiving a basal-bolus insulin regimen and having a longer duration of diabetes.

Notably, the number of severe hypoglycemic episodes reported across ONWARDS 1 to 5 was very low (8 events in total across ONWARDS 1-5 in the icodec arm). In the ≥65 years subgroup, there was only 1 reported severe hypoglycemic episode for icodec (ONWARDS 4) and 6 severe hypoglycemic episodes reported for OD comparators (ONWARDS 1-5). Elderly individuals are at a higher risk of adverse outcomes caused by severe hypoglycemia, such as cardiovascular events and death (21), and the results from this analysis suggest that icodec is a viable treatment option for older individuals, including those with a longer diabetes duration, such as the participants in ONWARDS 4. Similar findings have been reported for other basal insulin analogs such as insulin detemir, which showed similar efficacy and safety profiles in individuals with type 2 diabetes aged <65 years and ≥65 years when administered following physician-led dose titration (22).

Although differences in ethnicity and race are known to influence an individual's sensitivity and response to insulin therapy, no clinically meaningful differences in glycemic outcomes were observed across ethnicity and race subgroups in those receiving icodec vs OD comparator, irrespective of insulin experience (eg, insulin-naive individuals in ONWARDS 1, 3, and 5) or treatment regimen (eg, basal-bolus in ONWARDS 4) (4, 11). It should be noted that the trends identified in ONWARDS 4 suggest that change in HbA_1c_ in response to icodec or OD comparator might vary based on ethnicity (ie, in favor of icodec in the Hispanic/Latino subgroup); however, this trend was not substantially different from the remaining subgroup and may be influenced by disparities in the number of participants.

Here, we have presented the first data exploring the efficacy and hypoglycemia profile of a once-weekly insulin in different age, ethnicity, and race subgroups. A strength of this analysis is the inclusion of a robust, high-quality dataset derived from 5 phase 3a trials, based on a large multinational population of participants (n = 3765) covering 35 countries across North America, South America, Europe, and Asia. Therefore, the results are likely representative of a range of populations with type 2 diabetes. However, there are also some limitations associated with this analysis that warrant consideration. Across ONWARDS 1 to 5, there was a lower proportion of participants aged ≥75 years compared with other age subgroups. As a result, the generalizability of these specific results should be interpreted with caution. Additionally, there was an overrepresentation of participants in the White subgroup and low numbers of participants in the Black/African American subgroup in these trial populations, which may limit the robustness of the interpretation of the findings. Moreover, some differences in baseline characteristics were observed (eg, a lower BMI in the ≥65 years and Asian subgroups); therefore, residual bias in the endpoint estimates cannot be excluded. Participants in ONWARDS 5 used a dosing guide app to assist with icodec dose titration, and so it is not possible to distinguish between differences due to treatment or the app. Finally, observed discrepancies in endpoint estimate trends should be interpreted with caution owing to the reduced number of participants per subgroup.

Overall, in this post hoc analysis, the efficacy and hypoglycemia profile of icodec vs OD comparators was consistent across trial populations irrespective of age, ethnicity, or race, supporting icodec as a once-weekly treatment option for these individuals with type 2 diabetes. No blanket adjustments to the starting dose or titration of icodec would be expected to be necessary based on an individual's age, ethnicity, or race. As with other daily basal insulins, an individualized approach to treatment and diabetes management should be discussed between clinicians and individuals living with type 2 diabetes.

## Data Availability

Some or all datasets generated during and/or analyzed during the current study are not publicly available but are available from the corresponding author on reasonable request. Individual participant data will be shared in datasets in a de-identified or anonymized format. Shared data will include datasets from clinical research sponsored by Novo Nordisk and completed after 2001 for product indications approved in the EU and the USA. The study protocol and redacted clinical study report will be made available according to Novo Nordisk's data-sharing commitments. These data will be available permanently after research completion and after approval of product and product use in both the EU and the USA (no end date). Data will be shared with bona fide researchers submitting a research proposal requesting access to data for use as approved by the Independent Review Board (IRB) according to the IRB charter (see www.novonordisk-trials.com). These data can be accessed via an access request proposal form; the access criteria can be found at www.novonordisk-trials.com. The data will be made available on a specialized SAS data platform.

## Prior Presentation

Some data presented in this manuscript were previously presented at the 84th Scientific Sessions of the American Diabetes Association, Orlando, FL, USA, June 21-24, 2024, and at the 60th Annual Meeting of the European Association for the Study of Diabetes, Madrid, Spain, September 9-13, 2024.

## ClinicalTrials.gov Identifiers

ONWARDS 1 (NCT04460885); ONWARDS 2 (NCT04770532); ONWARDS 3 (NCT04795531); ONWARDS 4 (NCT04880850); ONWARDS 5 (NCT04760626).
